# Epistemic justice, African values and feedback of findings in African genomics research

**DOI:** 10.1080/11287462.2022.2124019

**Published:** 2022-09-21

**Authors:** Cornelius Ewuoso, Ambroise Wonkam, Jantina de Vries

**Affiliations:** a Steve Biko Centre for Bioethics, University of Witwatersrand, Johannesburg, South Africa; b Division of Human Genetics, University of Cape Town, Cape Town, South Africa; c McKusick-Nathans Institute and Department of Genetic Medicine, Johns Hopkins University School of Medicine, Baltimore, MD, USA; d Department of Medicine, Faculty of Health Sciences, University of Cape Town, Cape Town, South Africa; e Neuroscience Institute, University of Cape Town, Cape Town, South Africa

**Keywords:** African genomics research, solidarity, partiality, friendliness, African philosophy

## Abstract

This article draws on key normative principles grounded in important values – solidarity, partiality and friendliness – in African philosophy to think critically and deeply about the ethical challenges around returning individual genetic research findings in African genomics research. Precisely, we propose that the normative implication of solidarity, partiality and friendliness is that returning findings should be considered as a gesture of goodwill to participants to the extent that it constitutes acting for their well-being. Concretely, the value of friendliness may imply that one ought to return actionable results to participants even when their preferences regarding feedback are unknown. Notwithstanding, returning individual genetic results will have a cost implication. The cost of feeding back is relevant in the context of African genomics research projects, which are often funded by international sponsors and should be researched further.

## Introduction

An enduring ethical challenge in African genomics research relates to returning individual genetic research findings. Whilst there are important practical and financial questions about what should be fed back, an equally important consideration is whether, why and how African genomics researchers have obligations to return such results. It is tempting to merely interrogate international bioethics opinion and follow international best practices to answer this question. However, there is growing recognition in bioethics literature that in a quest toward decoloniality and epistemic justice, it is imperative that ethics recommendations and guidelines equally draw on the experiences, values, ways of being and becoming and philosophies of the people to whom they apply (Benatar & Brock, 2011). This is crucial since studies continue to demonstrate that individuals are more likely to abide by guidelines and policies that align with their values (Bardi & Schwartz, 2003; Schwartz & Bardi, 2001). For African health research, this implies an urgent need to interrogate how African philosophy informs on particular ethical challenges. Additionally, responsibilities arising from the moral norms espoused in African philosophy, ought to be clearly articulated and defended. This is important not just for the continent but also because “positive ethical insights from an African worldview … might enrich bioethics” globally (Barugahare, 2018, p. 98). Furthermore, drawing on African philosophy to derive ethical guidance for African genomics research could increase the likelihood that ethical recommendations would be broadly supported and implemented on the continent (Munung et al., 2021).

For this reason, in this article, we interrogate three key concepts in African philosophy that should inform reflection about researchers’ ethical obligations to return individual genetic research results in African genomics. These key concepts are solidarity, partiality and friendliness. Whilst we do not claim that these three concepts exhaust all key concepts in African philosophy, they nonetheless, occur frequently enough in the writings of African scholars to be considered good candidates for thinking critically about the ethical obligations around the return of individual genetic research results in ways that respond to the quest for epistemic justice (Ewuoso & Hall, 2019). Equally, the reader should notice how we understand African philosophy. With that term, we describe philosophical approaches that are mostly informed by intuitions, modes of experiencing the world, and ways of being and becoming that are predominant on the African continent and have not come to the region from elsewhere (Ewuoso & Hall, 2019). As remarked by Thaddeus Metz (Metz, 2010a, p. 50), “it is apt to call [this] moral theory … African because the ideas that it expresses and that inform it are much more salient there than in not only the West, but also the major Islamic and Hindu traditions.” Though solidarity, partiality, and friendliness are not unique to Africa, the thinking about these concepts in the writings of African scholars matches moral intuitions that are dominant in sub-Saharan Africa and have not come to the continent from elsewhere.

In subsequent sections, we first provide core descriptions of the concepts we draw on to consider the ethical challenges around returning individual findings. Second, we demonstrate their usefulness for addressing these challenges, implying that this section is primarily evaluative. Precisely, in the second section, we contend that the normative implication of solidarity, partiality and friendliness is that returning findings should be considered a gesture of goodwill to participants to the extent that it constitutes acting for their well-being. In the final section, we address a potential criticism that drawing on *three* concepts in a single article undermines our capacity to deeply interrogate those concepts and articulate the various ways African scholars have defended the concepts. In response, we demonstrate how this is not problematic.

## Solidarity

There are three connected salient components of solidarity in African philosophy, though we do not claim that these components exhaust the discussions on solidarity. They include (a) altruism or responsiveness to others for their sake; (b) engaging in mutual aid, and; (c) taking ownership/responsibility for one another’s destiny (Ewuoso et al., 2022; Metz, 2016). First, responsiveness to others for their sake often entails two components (empathy and sympathy), implying that it requires subjects of relationships to feel how objects of relationships are likely to feel when they are not aided, and to acknowledge objects who require their (subjects’) aid and the ways they do. Second, the obligation of mutual aid is often expressed by the African maxim of Ubuntu: “I am because we are, we are, therefore, I am”. It informs agricultural practices like *Letsema* whereby members assist one another to harvest their crops. Finally, collective responsibility is grounded in the idea that human lives are deeply interconnected. African philosophy suggests that solidarity is a core component of showcasing humanity or becoming a person. For instance, consider the following remark about solidarity by Thaddeus Metz:

… there is reference to a relationship of solidarity, achieving the good of all, being sympathetic, acting for the common good, serving others and being concerned for others’ welfare. Here, too, there is a behavioural component, of doing what is likely to enable others to live better lives, [and] a psychological one, of doing so consequent to sympathy and for the sake of the other. In sum, the more one prizes these kinds of other-regarding tendencies, or people’s capacity for them, the more humanness one exhibits or the more of a person one is (Metz, 2016, p. 138).

## Partiality

The emphasis on acting in ways that promote solidarity in African philosophy is qualified by the nature of the relationship between actors. In other words, the obligation of solidarity is a qualified one. Partiality in African scholars’ writings tends to imply that one has a stronger obligation towards current, actual and longstanding relationships. The relevant saying is “family first, and charity begins at home” (or blood is thicker than water). This maxim implies differentiated obligations towards different people based on the duration, depth and nature of the relationship one has with them. Where there is some relationship, there is some obligation – the closer the relationship, the stronger the obligation. The obligations to those with whom one has no relationship are limited by the obligations one has towards those with whom there is a stronger relationship. Thus, the obligation to act for the sake of others, engage in mutual aid or assume responsibility for the destinies of one another is strongest where there are sufficiently close proximities, existing ties, longstanding relationships such as familial relationships, and weakest where no relationships exist.

## Friendliness

Friendliness is also a core value. For instance, take the following remark by the Nobel Peace Prize winner Desmond Tutu, “harmony, friendliness, community are great goods. Social harmony is for us the *summum bonum* – the greatest good. Anything that subverts or undermines this sought-after good is to be avoided like the plague” (Tutu, 1999, p. 63). Friendliness is a combination of goodwill (that is, acting to improve others’ well-being) and identification (that is, thinking of oneself as part of a “we”). Both require one to emotionally, cognitively and behaviourally share a way of life with others and act in ways that promote their well-being. The African ethics of friendliness requires individuals to be friendly to those who have been friendly and to exhibit proportional unfriendliness to individuals who act in an unfriendly manner.

## Normative implications for african genomics research

There are many normative implications of African thinking about solidarity, partiality and friendliness for various questions regarding the return of individual genetic research findings in African genomics research. For instance, one implication is that it may require one to return findings to participants if it improves their quality of life on the balance of probabilities. This implication is grounded in the obligation to act or be responsive to others for their sake. One way of being responsive to others for their sake is acting in ways that are likely to improve their quality of life. Suppose a researcher discovers a clinically actionable finding. In that case, indifference to others’ well-being (that is, the well-being of participants) or the failure to improve their quality of life when one could, entails a failure to be *responsive* to others or exhibit solidarity. In African philosophy, quality of life is not limited to actual health improvement but includes the capacity to relate with others, live communally or develop one’s humanness/personhood (Ewuoso & Hall, 2019). Accordingly, a finding has positive implications for one’s quality of life if it could potentially improve one’s health, the capacity to relate with others and/or develop one’s humanness/personhood through communal relationships. For example, a finding of misattributed paternity may not be clinically actionable but could potentially provide the knowledge of the family where one ought to develop their personhood.

A critic will be right to point out here that the ways individuals acquire identity and personhood in African scholarship are more complex than we have described. Precisely, it tends to be the case that identity and personhood are acquired through various means that are not limited to socialization, adoption, biological birth, and interconnectedness, to name a few. The knowledge of one’s parentage may not be sufficient to gain personhood. Adopted babies, including children who falsely assume a genetic relationship to a parent, could still acquire personhood in families and cultures that adopt them supposing they act in the appropriate ways (Eze, 2014; Idang, 2015; Kanneh, 1998). In fact, for one author, culture – rather than a biological relationship – is what defines an individual’s identity and personhood (Idang, 2015). Though one of us addresses ethical issues around misattributed paternity in a different article (Ewuoso, 2020a), it is nonetheless important to acknowledge here that a finding of misattributed paternity could equally be deeply disturbing to individuals and divisive to family and community, that is, it could have unintended consequences at the individual, family and community levels. Consequently, our claim here is not that misattributed findings generate genuine dilemmas and how they do or that *all* findings of misattributed parentage ought to be returned. Rather, our claim here is that a finding will have different significance, implying that a finding will affect one’s quality of life in various ways, including clinically and personally. Suppose this is true, what this application of the principle of solidarity suggests is that future research should specifically interrogate and outline how specificfindings will improve an individual’s quality of life. For example, there are ongoing efforts to describe all disease-causing gene variants or the types of genetic variants that are associated with known diseases (Claussnitzer et al., 2020; Emilsson et al., 2008; Fieggen & Ntusi, 2019). The obligation to assume responsibility for each other’s destiny will support these efforts and equally require that when such disease-causing gene variants are discovered during research, a researcher or research team ought to return such findings to participants. This will also be a good way to reciprocate participants for participation in research (Ralefala et al., 2020). Notice here that we do not claim that this is the only way to reciprocate participants. Evidently, there are many other ways to reciprocate participation, including returning aggregate findings that improve community health, and the obligation of solidarity is not unqualified. In a subsequent paragraph, we describe how this obligation may be limited.

Similarly, the value of friendliness may imply that one ought to return an actionable result – through means that are not unfriendly – to participants even when their preferences regarding feedback are unknown. The thinking here is that we have a primary obligation to end unfriendliness – in which individuals have instead been self-interested, sought to think of themselves as an “I” rather than a “we”, act in ways that undermine their well-being or failed to care about others behaviourally – before promoting new friendships (Metz, 2010b). In many African cultures, elders in communities are often typified as dignified and wise figures responsible for guiding young ones and urging them to act in friendly ways. The authorities of the elders equally extend to compelling individuals to act in relevant ways. As repositories of moral knowledge, elders can make judgements about instances where individuals have failed to act for the well-being of others. Notice that the preceding does not imply that only elders enjoy this privilege. Depending on the hierarchical structure of the traditional society, this privilege may rest in the monarch or a council of consultors (or men if it is a highly patriarchal culture) (Aiyedun & Ordor, 2016; Mbele, 2004; Michel et al., 2020).

In African scholarship, illness and disease are conditions that undermine friendliness or one’s capacity for it. As previously stated, friendliness tends to be considered as an important good in African philosophy. Consequently, the value of friendliness and the emphasis placed on the same imply that research primarily (and not exclusively) ought to interrogate and address diseases that currently plague humans or undermine the capacity to share a way of life before seeking out, say (new) forms of enhancement or interventions. Concretely, suppose there is a primary obligation to end unfriendliness or end acts that undermine friendliness. In that case, research ought to seek out interventions to eliminate diseases like HIV and the corona or monkeypox viruses that currently undermine human relationships or the opportunities to enjoy deep human interactions before seeking out non-therapeutic enhancements for instance. Within the context of African genomics, the value of friendliness could imply prioritizing studies that address Africa’s disease burden. It will equally imply prioritizing research on strains of virus or diseases that plague the continent. From the perspective of friendliness, research ought to have a social value or relevance to the host community (MacKay, 2016).

Someone may ask here, are all acts where a person thinks of himself as “I” rather than a “we” necessarily impermissible? Genomic studies are often expensive and require external sponsors. Consider a researcher who fails to make any research progress, address promised objectives or spends without giving any report. Should a sponsor continue to fund such a project because this would be “unfriendly” (even if there are serious consequences like the loss of money to the sponsor), and thus impermissible? Given the emphasis on the *combination* of identification and goodwill in African philosophy, then the thinking of oneself as an “I” rather than a “we” appears to be an instance of unfriendliness. But not *all* unfriendliness is necessarily impermissible. Infringements of rights through involuntary admission or quarantine are other forms of unfriendliness that may be permissible to end comparative unfriendliness (sickness caused by the spread of a virus, since sickness undermines an individual’s capacity for relationship). The value of showcasing humanity through friendliness requires one to be friendly to the extent that one can oneself and to use unfriendliness only when necessary to end proportional unfriendliness (Ewuoso, 2020b). Suppose one fails to be friendly, such as when a researcher fails to deliver on promises or justify research spendings. In this regard, others (like the researcher’s institution) have a duty to step in and end such unfriendliness if the sponsor has not taken action to end it. In fact, in a broader African thinking of friendliness, this could justifiably be interpreted as upholding friendliness because the researcher is a friendship denier and continuing to befriend one is to deny the value of friendship. It could also be interpreted as using unfriendliness. When interpreted in the latter sense, the unfriendliness ought to be proportional. This seems to be Metz’s point as evidenced in the following remark:

Suppose now that you refuse to end your unfriendliness and continue to mistreat your friend (whom we presume does not warrant it by virtue of having been unfriendly herself). If a third party were in a position to force you to stop … he would be justified indoing so … he would not be [dishonouring] the value of friendship in doing so, just the opposite (Metz, 2010b, p. 92).

Notwithstanding, the obligation to feedback individual genetic research results is not absolute and may be limited by the nature and relative depth of the relationship. In this regard, partiality can enhance our thinking about the limits of the duty to return results. Of interest here is that there may be varying types of relationships between African genomics researchers and participants. Namely, most African genomic studies focus on health and – at least in H3Africa – tended to be conducted by clinicians in clinical settings. In those cases, half of the participants are not just that; they are also patients with whom the clinician-researchers and the broader research teams may have built a long-lasting relationship. Contrarily, such a relationship may not exist with other participants who are recruited as controls completely outside of clinical settings or an established treatment relationship. The same would be true for non-medical genomics research projects like anthropological genetic projects. What partiality would suggest is that the obligation to return individual results depends on the intensity of the relationship between the researcher and participant; the more intense or enduring that relationship is, the greater the researchers’ obligation to return findings. In cases where there is an established relationship, researchers arguably may have a greater obligation to return results than in cases where there is not.

But partiality also compels us to explore the nature of the relationship between actors, that is, between researchers and participants. In genomics research, the nature of the shared experience is arguably a joint commitment to advance science, such as through the contribution of generalizable knowledge. Accordingly, the obligation to return actionable findings may be limited by the more significant obligation to advance science; if feeding back individual results endangers this obligation, then they may not be fed back. This obligation may also be limited by other deeper relationships the researcher may have towards others like the research institution or funders. From the African philosophy perspective, it cannot be reasonably asked that the researcher feeds back results if this will undermine their capacity to fulfil other more significant obligations.

Finally, the obligations we describe here add something new to international best practice in (African) genomics. Precisely, they contribute to how under-explored concepts in African philosophy can enhance our thinking about *what constitutes* best practice in African genomics and thinking about the ethical issues around ethical challenges generated by returning individual genetic research findings on the continent. In this regard, it is a new way of thinking about returning individual genetic research findings. As previously stated, this is important for epistemic justice. Notably, within the context of African genomics, it matters that concepts and values informed by intuitions and ways of becoming dominant on the continent are acknowledged as primary contributors to the knowledge production, which underlies frameworks or approaches we draw on to think critically about issues in African genomics.

## Potential objections

We have explored some objections in the previous sections. One more objection is worth considering. Precisely, a critic may point out that the approach to draw *on three* concepts to think critically about the ethical challenges around returning individual genetic research findings in African genomics research undermines our capacity to, (i) explore deeply the various ways scholars in African philosophy have formulated each concept, (ii) lucidly outline the various norms that can emerge from such formulations, and (iii) carefully demonstrate how these norms are useful for thinking about challenges around returning individual genetic research findings. For this reason, it would be preferable to draw on only one concept, describe the various formulations of the same and the moral norms to which the concept gives rise, and demonstrate their implications.

In response, we acknowledge that *descriptive* studies (like systematic and scoping reviews) will be required to outline the various formulations (in African philosophy) of the concepts we draw on to address the critical question in this article. Nonetheless, we do not think this is problematic since the description we provide appears to be the views of many scholars in African philosophy as demonstrated by the various studies (Ewuoso & Hall, 2019; Ewuoso et al., 2022; Metz, 2009; Molefe, 2016) and is sufficient to undertake the mostly e*valuative* aim of this article, which is, to demonstrate how the common thinking about the concepts we draw on can enhance our knowledge of what constitutes best practice regarding individual genetic research findings in African genomics. There is another benefit of this approach that is directly relevant to this potential criticism. Broadly, there are many ethical challenges around returning individual genetic research findings, and it is not *always* possible to identify one concept that will be useful to address *all* the challenges that returning findings raises. For example, friendliness might be useful for thinking about *why* clinically actionable findings ought to be returned. But it is not useful for thinking about the *limits* of the duty (if any) to return. Given that the article’s primary focus is to think deeply about the challenges that returning findings raise, it seems that it is a better approach to draw on – within what is reasonable – many concepts that can help us interrogate the issue in-depth.

## Concluding remarks

Taking the previous paragraphs on board, solidarity, partiality, and friendliness imply that there is at least a partial obligation to feedback findings that meet the threshold for return to participants. Additionally, it may equally be a gesture of goodwill to return findings to relatives of participants to the extent that the researcher is acting for their well-being. Empirical studies support this normative implication (Ewuoso, 2016). However, this partial obligation to return individual genetic results will have a cost implication. Precisely, we acknowledge that the obligations entailed by solidarity, friendliness and partiality will undoubtedly raise many questions regarding who ought to fund feedback of findings. Though we address ethical challenges around cost, as well as how those challenges may be addressed, in a different and mostly normative study (Ewuoso, Berkman, Wonkam, & de Vries, 2022). Nonetheless, This question concerning the cost of feeding back is relevant within the context of African genomics research projects, which are often funded by international sponsors. Suppose friendliness implies that researchers ought to return actionable findings. In that case, who ought to fund such feedback? This question is especially important for findings that have not been anticipated in research budgets. Here, cost is not limited to validation, necessary counselling, and foreign expertise especially for contexts where it would be necessary to access such a critical mass of expertise because genetic diagnostic laboratories are unavailable and hospitals and/or institutions where research is conducted are not well-resourced. There may also be downstream costs like follow-up tests and health care (Papaz et al., 2019). As we continue developing African genomics research, more studies are required in this space to increase our understanding concerning the cost of returning individual genetic research results within the context of African genomics research and *who* should fund feedback. However, the African view of collective responsibility suggests a cost-participative model, whereby researchers contribute their expertise and sponsors and institutions dedicate funds or personnel to mitigate cost. Concretely, this could imply that research institutions dedicate a department that provides genetic counselling to participants who require such service. The relevant maxim here is “it takes a village to raise a child.” Nonetheless, similar to the thinking about other key concepts, the moral norms entailed in the cost-participative model are not absolute. In other words, the duty to contribute towards mitigating the cost of feeding back findings is only a *prima facie* duty and is limited by the greater obligation to advance science.

Research is equally required to increase our understanding of *whether* and *how* context can inform what finding is actionable and returnable (Wonkam & de Vries, 2020). Why is this important within the context of African genomics research? First, actionability tends to imply the availability of preventive measures. Yet many hospitals to which African participants have access are overburdened and/or under-resourced to manage diseases. Second, the concept equally entails the idea that participants will be able to pull resources together to act upon the communicated information. Yet, most genomic research studies in Africa occur in communities that are sometimes burdened by poverty. Third, actionability appears to rely on a list of reportable findings such as the one developed by the American College of Medical Genetics and Genomics (ACMG). Yet disease burden in Africa tends to differ from those elsewhere. For instance, absent from the ACMG list are pathogenic mutations like sickle cell disease mutations that are common in some African populations.

Finally, actionability appears to connote the idea of clinical relevance. Yet, Africa’s dominant value (of communal relationships) implies that a finding will be actionable even though it is only of personal relevance, such as when the finding can foster a participant’s capacity to relate with others in the community. This final point implies that African values have practical relevance for thinking about *what* to return. Hence, in some way, this commentary is epistemic justice in action, decentreing knowledge production in African genomics research by contributing knowledge from Africa to influence research in Africa and increasing our understanding of what the obligation – that is informed by an African voice – to return individual genetic research finding looks like.

AW and JdV are supported by the IFGENERA H3Africa ELSI Collaborative Centre grant, awarded by the National Human Genome Research Institute of the National Institutes of Health under Award Number U54HG009790. The content is solely the responsibility of the authors and does not necessarily represent the official views of the National Institutes of Health. The authors are also grateful to the reviewers for their insightful comments.
